# Flightless I exacerbation of inflammatory responses contributes to increased colonic damage in a mouse model of dextran sulphate sodium-induced ulcerative colitis

**DOI:** 10.1038/s41598-019-49129-6

**Published:** 2019-09-05

**Authors:** Z. Kopecki, G. Yang, S. Treloar, S. Mashtoub, G. S. Howarth, A. G. Cummins, A. J. Cowin

**Affiliations:** 1 Regenerative Medicine, Future Industries Institute, University of South Australia, Mawson Lakes, Adelaide, South Australia Australia; 2 School of Pharmacy and Medical Sciences, University of South Australia, Adelaide, South Australia Australia; 3 School of Animal and Veterinary Sciences, The University of Adelaide, Roseworthy, Adelaide, South Australia Australia; 4 Department of Gastroenterology, Women’s and Children’s Hospital, North Adelaide, South Australia Australia; 5 Discipline of Physiology, Adelaide Medical School, The University of Adelaide, Adelaide, South Australia Australia; 6 Department of Gastroenterology and Hepatology, The Queen Elizabeth Hospital, Woodville South, Adelaide, South Australia Australia

**Keywords:** Chronic inflammation, Ulcerative colitis

## Abstract

Ulcerative colitis (UC) is a chronic inflammatory bowel disease characterized by cytokine driven inflammation that disrupts the mucosa and impedes intestinal structure and functions. Flightless I (Flii) is an immuno-modulatory protein is a member of the gelsolin family of actin-remodelling proteins that regulates cellular and inflammatory processes critical in tissue repair. Here we investigated its involvement in UC and show that Flii is significantly elevated in colonic tissues of patients with inflammatory bowel disease. Using an acute murine model of colitis, we characterised the contribution of Flii to UC using mice with low (*Flii*^+/−^), normal (*Flii*^+/+^) and high Flii (*Flii*^*Tg/Tg*^). High levels of *Flii* resulted in significantly elevated disease severity index scores, increased rectal bleeding and degree of colon shortening whereas, low *Flii* expression decreased disease severity, reduced tissue inflammation and improved clinical indicators of UC. Mice with high levels of *Flii* had significantly increased histological disease severity and elevated mucosal damage with significantly increased inflammatory cell infiltrate and significantly higher levels of TNF-α, IFN-γ, IL-5 and IL-13 pro-inflammatory cytokines. Additionally, *Flii* overexpression resulted in decreased β-catenin levels, inhibited Wnt/β-catenin signalling and impaired regeneration of colonic crypts. These studies suggest that high levels of Flii, as is observed in patients with UC, may adversely affect mucosal healing via mechanisms involving Th_1_ and Th_2_ mediated tissue inflammation and Wnt/β-catenin signalling pathway.

## Introduction

Ulcerative colitis (UC) is a chronic inflammatory bowel disease (IBD) with incidences of 7.6 to 13.9 cases per 100,000 people in Westernised industrialized nations. It is defined as a lifelong condition with periods of remission, which manifests in bloody diarrhoea, mucus and abdominal pain^1^. It peaks in young adults and to lesser extent in the elderly. Pathogenesis of UC is unknown, although genetic susceptibility, environmental factors, microorganisms, immune dysregulation and chemical mediators have all been suggested as possible contributing factors^2^. Symptoms may relapse and remit, but mucosal inflammation continues with spontaneous remission being uncommon. Treatment includes corticosteroids, aminosalicylates, immunomodulators and biologics such as anti-tumour necrosis factor-α (TNF-α) antibody, and surgical resection^3^. Randomised controlled trials have demonstrated that infliximab and adalimumab, TNF-α antibody therapies, are effective for patients with moderate to severe colitis significantly improving mucosal healing and rates of disease remission hence decreasing the need for colectomy^3,4^. However, access and cost of this therapy is still a limiting factor for many UC patients, highlighting the need for novel targeted therapies. Additionally, only about two-thirds of subjects with UC respond well to treatment and in severe disease with pancolitis there is a cumulative risk of colon cancer that increases with time due to chronic inflammation.

Mucosal healing is currently accepted as a critical endpoint in the management of UC and regulation of colonic inflammation underpins mucosal healing. Many of the current UC treatments achieve clinical remission without complete mucosal healing which has been demonstrated to correlate with reduced risk of relapse and hospitalisation^5^. Immuno-modulatory protein Flightless I (Flii) impairs skin barrier development, function and recovery post skin blistering and wounding^6,7^. It negatively affects cellular processes including cellular adhesion, migration and proliferation as well as tight junction formation and macrophage and fibroblast cell secretion of TNF-α in the context of wound healing^7–12^. Reduced levels of Flii expression, both genetically and using Flii neutralising antibodies, improves skin repair and regeneration in both small and large animal models of healing^7,8,10–12^. Studies have identified Flii as a repressor of estrogen receptor signalling and apoptosis suggesting roles in promotion of both skin cancer and breast cancer progression^13–15^. In contrast, Flii positively influences tissue regeneration in the context of Wnt signalling pathways during hair follicle regeneration and claw and digit tip regeneration^16,17^ illustrating the diverse roles of this cytoskeletal protein. Importantly, Flii modulates TLR-4 mediated inflammatory responses^18,19^, augments Th_1_/Th_2_ cell responses as well as autoantibody production and regulates inflammation in a number of inflammatory skin conditions including psoriasis, atopic dermatitis and epidermolysis bullosa acquisita^20–24^. Its function has not been investigated in the intestine.

Using human UC samples, we sought to examine the levels of Flii in human disease. Additionally, using the dextran sulphate sodium (DSS)-induced colitis model in mice, a reproducible and well documented model of large intestinal damage^25^, in conjunction with mice genetically manipulated to have either high or low levels of Flii, we investigated the role of Flii in UC and mucosal damage. Lastly, we aimed to characterise the effects of altered Flii levels on tissue inflammation in this model of DSS-induced colitis. Our hypothesis was that Flii would alter tissue inflammation and promote colitis development.

## Results

### Flightless I is significantly increased in human UC colonic tissue

**Figure 1:**
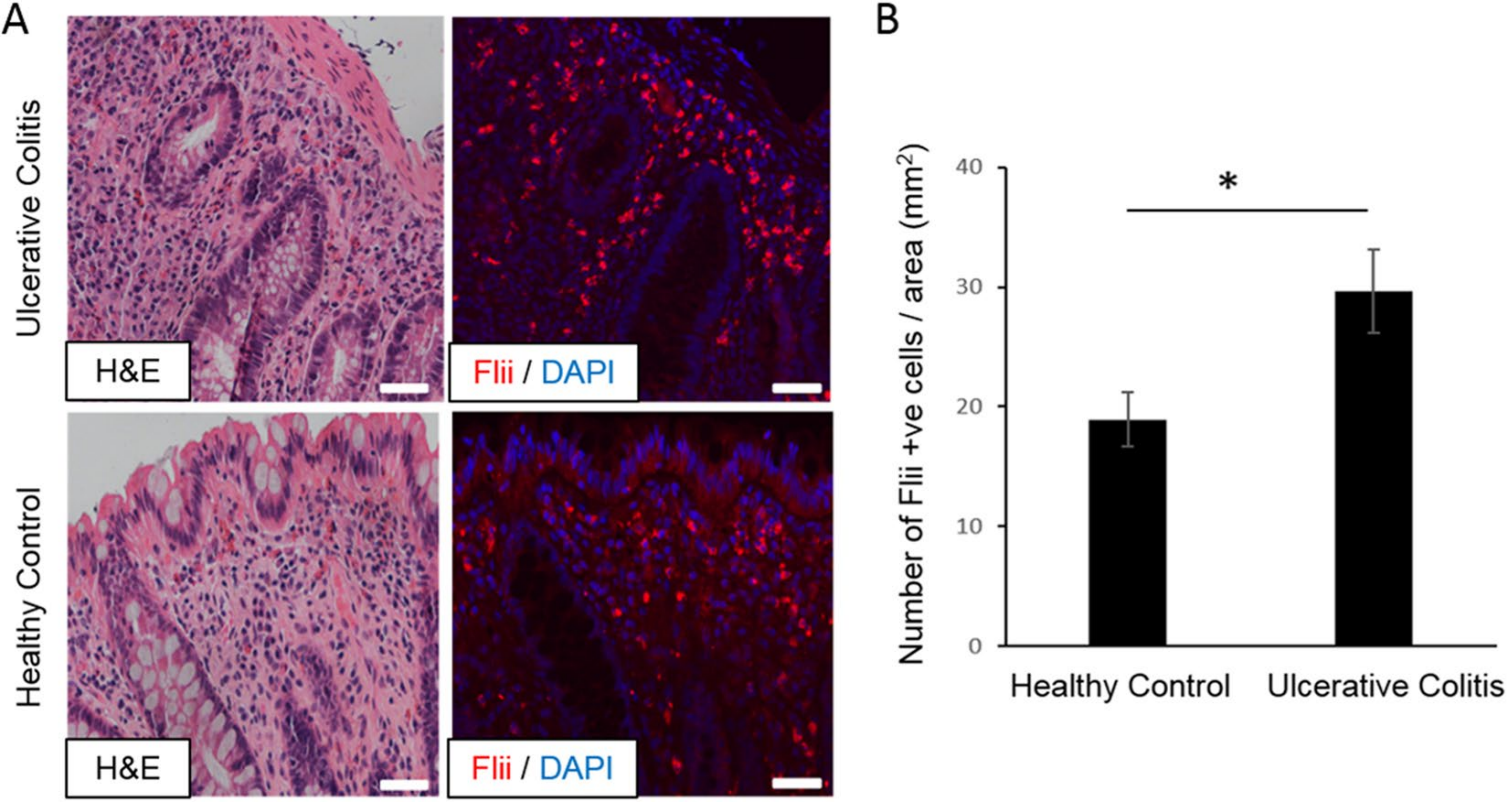
Significantly increased number of Flii positive cells were observed in colonic tissue sourced from UC patients. (**A)** Representative H&E and Flii stained sections of colonic tissue from UC patients and healthy control patients. UC patients have a clear increase in inflammatory cell infiltrate and increased numbers of Flii positive inflammatory cells (red) in lamina propria compared to healthy control tissue. DAPI nuclear marker is stained blue. Magnification × 20. Scale bar = 50 µms. (**B)** Graphical representation of numbers of Flii positive cells in lamina propria of UC patients compared to healthy control patients. Mean ^+/−^ SEM. *p < 0.05.

Histological analysis of human samples revealed classic morphological characteristics associated with UC including increased inflammatory infiltrate in lamina propria, crypt distortion and shortening when compared to healthy control (Fig. 1A). Inflammatory infiltrate included neutrophils with infiltration of crypts and formation of crypt abscesses (not shown). Crypts were shortened and separated from muscularis mucosae with occasional branching (not shown) indicating chronicity of poorly controlled disease. No Flii staining was observed in colonocytes lining the lumen or colonic crypts. However strong Flii staining was observed in the inflammatory infiltrate present in the lamina propria (Fig. 1A). Cell counts of Flii positive cells in the human colonic tissue revealed significantly elevated levels of Flii in UC patients compared to healthy controls (Fig. 1B). Isotype control staining revealed negligible fluorescence (data not shown).

### Overexpression of Flightless I increases clinical disease severity following DSS consumption

**Figure 2:**
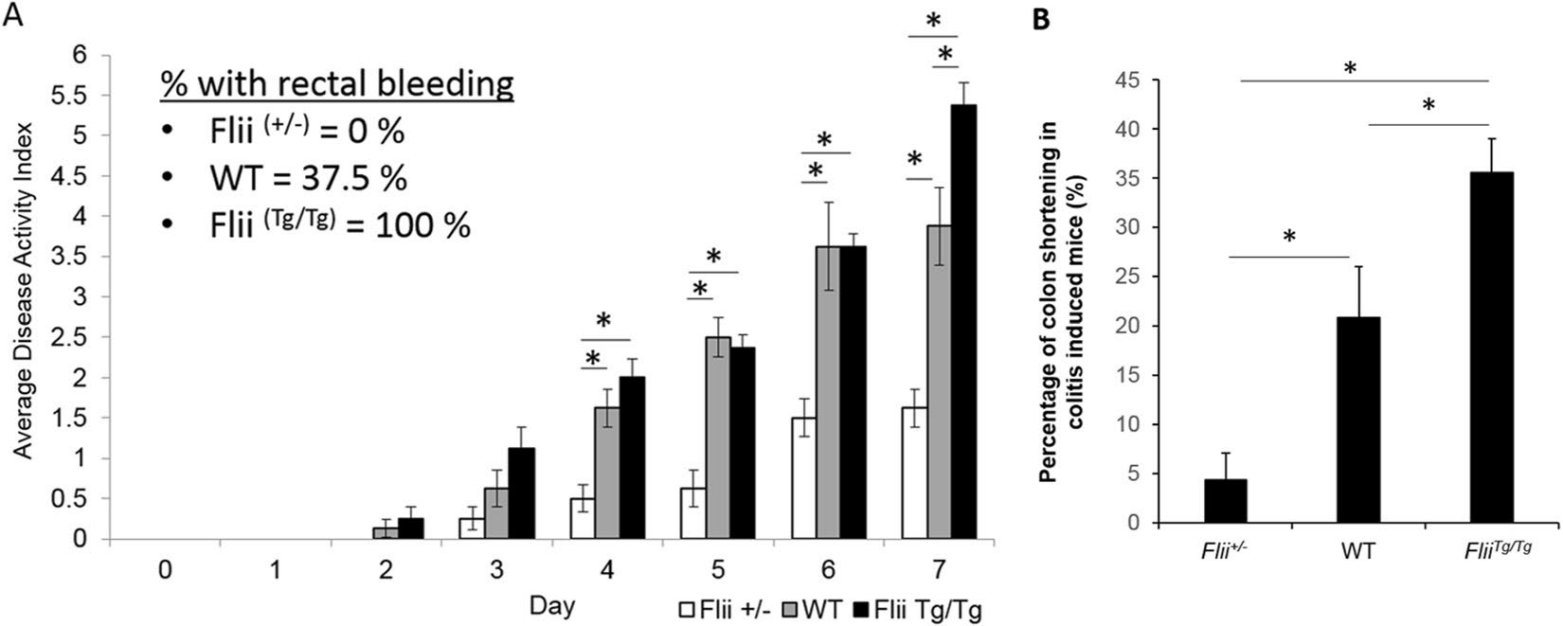
Increased clinical disease severity is observed in *Flii* overexpressing mice following DSS consumption. (**A)** All *Flii* overexpressing mice showed evidence of rectal bleeding and a significantly increased disease activity index on day 7 of the experiment compared to wild-type mice. *Flii* deficient mice show no evidence of rectal bleeding and significantly decreased disease activity index from day 3 of the experiment. (**B)** Colon lengths of colitis-induced *Flii*^+/−^, wild-type and *Flii*^*Tg/Tg*^ mice were compared to colon lengths in water control counterpart mice, and percentage change in colon shortening analysed. Colitis-induced *Flii*^+/−^ mice have significantly reduced percentage of colon shortening while *Flii*^*Tg/Tg*^ have significantly increased percentage of colon shortening compared to wild-type counterparts, suggestive of increased UC damage in mice with high Flii levels. n = 8/genotype. Mean ^+/−^ SEM. *p < 0.05.

While only 37.5% of wild-type mice exhibited evidence of rectal bleeding, 100% of Flii overexpressing mice (*Flii*^*Tg/Tg*^) showed signs of rectal bleeding and all had significantly higher average disease activity index on day 7 compared to either *Flii*^+/−^ mice or wild-type counterparts (Fig. 2A). In contrast, mice with low Flii (*Flii*^+/−^) exhibited no rectal bleeding and significantly decreased average disease activity index from day 3 of the experiment (Fig. 2A). Examining the visceral and gastrointestinal organ weights showed no significant differences between the three genotypes (data not shown). However, when we compared the degree of colon shortening in colitis-induced mice by analysing the change in colon lengths in colitis-induced vs water control mice results showed that colitis-induced *Flii*^+/−^ mice have reduced percentage of colon shortening compared to wild-type counterparts while *Flii*^*Tg/Tg*^ mice had significant increase in percentage of colon shortening compared to both *Flii*^+/−^ and wild-type counterparts suggestive of greater disease severity in response to higher levels of Flii (Fig. 2B).

### Increased histological disease severity is observed in colitis-induced *Flii*^*Tg/Tg*^ mice

**Figure 3:**
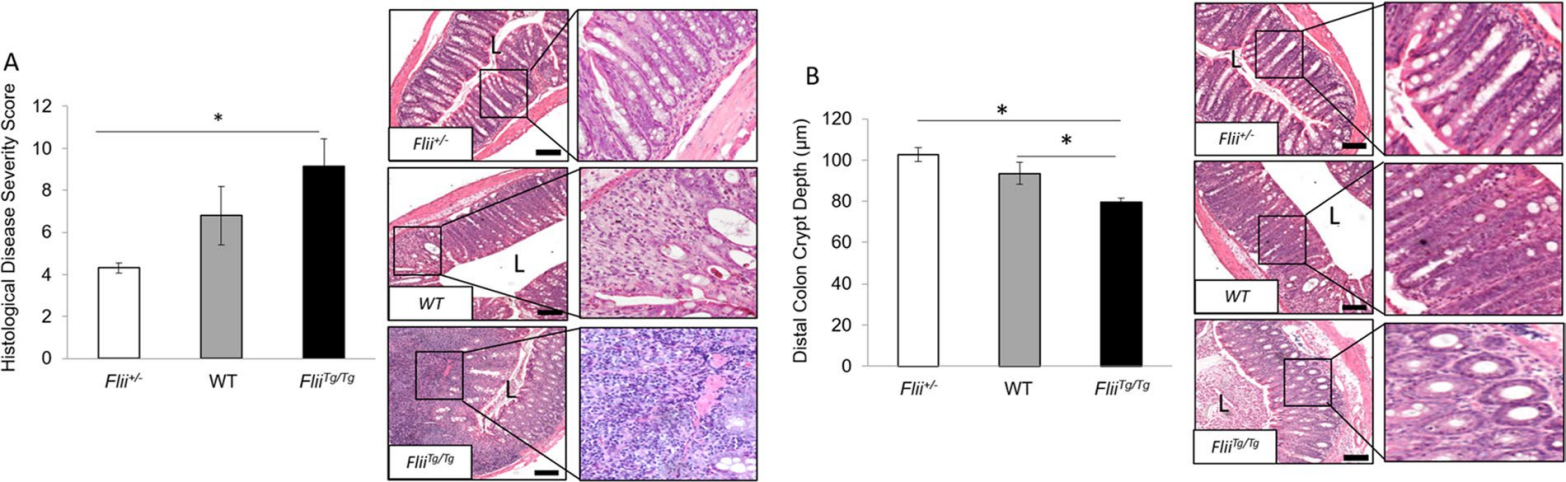
Increased disease histological severity is observed in *Flii* over-expressing mice. (**A**) *Flii* overexpressing mice showed significantly higher histological disease severity compared with *Flii* deficient mice, with significantly damaged mucosal tissue and high level of pro-inflammatory cell infiltrate. (**B)** *Flii* overexpression resulted in significantly delayed healing of blistered mucosal tissue with significantly reduced distal colon crypt depth compared to both wild-type controls and *Flii* deficient mice counterparts. Magnification ×4 and ×20. Scale Bar = 200 µm. n = 8/genotype. Mean ^+/−^ SEM. *p < 0.05.

**Figure 4:**
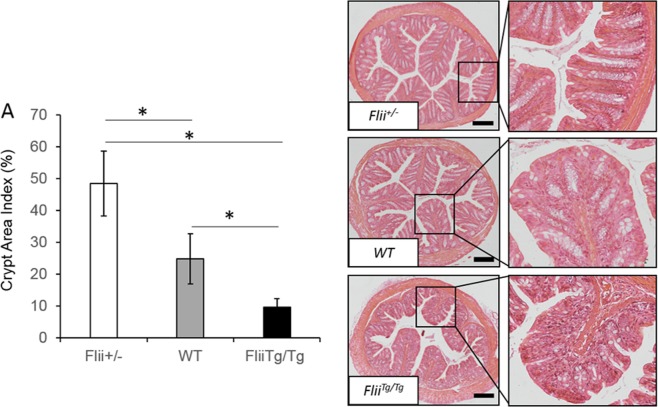
High Flii levels promote mucosal damage in DSS model of UC. **(A)** *Flii* overexpressing mice show significantly decreased crypt area index indicative of increased disease severity compared to wild-type controls, while Flii deficient mice have significantly increased crypt area index indicative of decreased mucosal blistering and gut damage in the DSS model of UC. n = 8/genotype. Magnification ×4 and ×20. Scale Bar = 200 µm. Mean ^+/−^ SEM. *p < 0.05.

Distal colonic tissue from *Flii*^+/−^, wild-type and *Flii*^*Tg/Tg*^ colitis-induced animals was examined and a clear increase in colitis severity was observed in mice with elevated levels of Flii including elevated polymorphonuclear infiltration (Fig. 3A). Overall there was a statistically significant increase in histological disease severity between *Flii*^*Tg/Tg*^ colitis-induced animals compared to *Flii*^+/−^ counterparts (Fig. 3A). Additionally, colitis-induced *Flii*^*Tg/Tg*^ mice showed significantly delayed healing of damaged mucosal tissue as demonstrated by significantly reduced distal colon crypt depth compared to both *Flii*^+/−^ and wild-type mice counterparts (Fig. 3B). Evidence of increased disease histological severity in colitis-induced *Flii*^*Tg/Tg*^ mice was also observed following analysis of crypt area index revealing a significantly decreased crypt area index in *Flii*^*Tg/Tg*^ mice compared to *Flii*^+/−^ and wild-type mice counterparts (Fig. 4). Conversely, mice with low levels of Flii showed a significant increase in crypt area index compared to both normal and *Flii*^*Tg/Tg*^ mice suggestive of decreased colitis severity.

### Decreased *Flii* levels lead to a reduced inflammation in DSS-induced colitis

**Figure 5:**
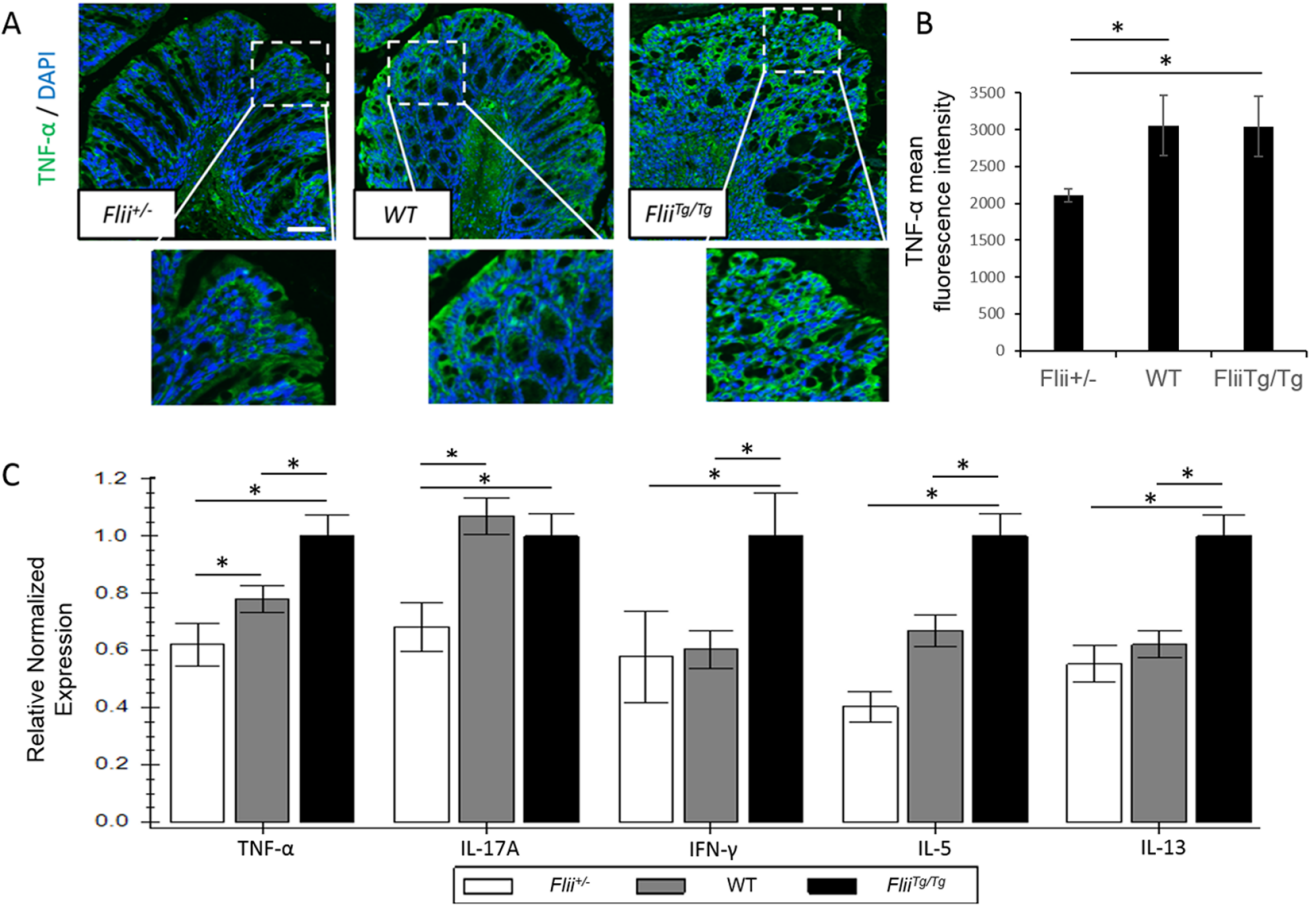
Flii regulates immune responses in a DSS-induced model of UC. (**A,B)** Representative images and graphical analysis of TNF-α levels in distal colon of colitis-induced *Flii*^+/−^, wild-type and *Flii*^*Tg/Tg*^ mice. TNF-α levels were predominantly present in apical enterocytes and significantly lower levels were observed in *Flii*^+/−^ colitis-induced mice. Magnification ×10. Scale Bar = 100 µm. (**C)** Overexpression of *Flii* increases distal colon inflammation with significant upregulation of both Th_1_ and Th_2_ pro-inflammatory cytokine profiles while *Flii* reduction decreases inflammatory responses in a DSS-induced mouse model of UC. n = 6. Mean ^+/−^ SEM. *p < 0.05.

Flii is a known regulator of cellular proliferation and inflammation^6,9,10^. To ascertain the effect of differential *Flii* on mucosal healing of colitis-induced mice, enterocyte proliferation and total tissue inflammation were assessed. No effect of Flii altering levels were observed on enterocyte proliferation as demonstrated by analysis of the numbers of PCNA positive cells in the crypts of colitis-induced *Flii*^+/−^, wild-type and *Flii*^*Tg/Tg*^ mice (Supplementary Fig. 1A). However, assessment of total tissue inflammation by MPO analysis revealed significantly decreased levels of tissue inflammation in colitis-induced *Flii*^+/−^ mice compared to both wild-type and *Flii*^*Tg/Tg*^ counterparts (Supplementary Fig. 1B). Additionally, distal colons of colitis-induced *Flii*^+/−^ mice showed significantly lower levels of TNF-α compared to wild-type and *Flii*^*Tg/Tg*^ counterparts with staining observed in only apical enterocytes (Fig. 5A,B). Increased levels of Flii also resulted in exacerbation of both Th_1_ and Th_2_ immune responses in colitis-induced mice with significantly greater levels of TNF-α, IFN-γ, IL-5 and IL-13 (Fig. 5C) being observed. Similarly, mice with low Flii exhibited a reduced inflammatory response in their distal colons with significantly decreased levels of TNF-α, IL-17A and IL-5 (Fig. 5C).

### *Flii* over-expression inhibits Wnt/β-catenin signalling and impairs regeneration of colonic crypts in DSS-induced colitis

**Figure 6:**
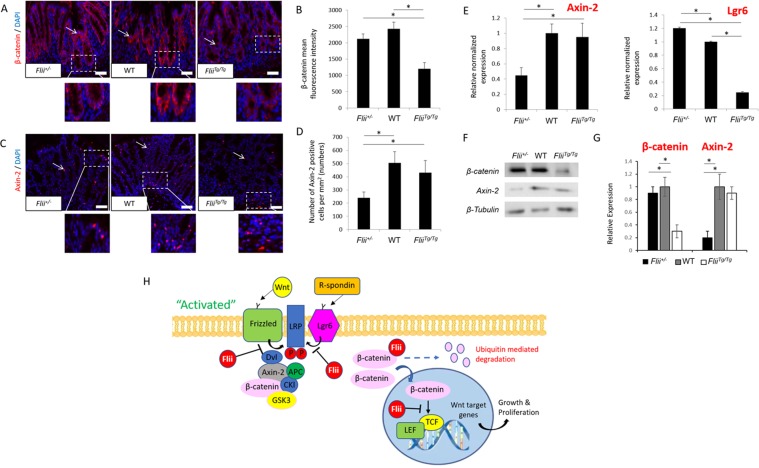
*Flii* over-expression inhibits Wnt/β-catenin signalling in DSS-induced colitis. **(A–D)** Representative images and graphical analysis of β-catenin and Axin-2 levels in distal colon of colitis-induced *Flii*^+/−^, wild-type and *Flii*^*Tg/Tg*^ mice. β-catenin staining was most prominent at the base of colonic crypts while punctate Axin-2 staining was detected throughout the colonic epithelium. *Flii* overexpression resulted in decreased β-catenin levels while Flii deficiency resulted in decreased Axin-2 levels. Magnification ×20. Scale Bar = 50 µm. n = 6. Mean ^+/−^ SEM. *p < 0.05. (**E)** Effects of Flii gene expression on Axin-2 and Lgr6 mRNA levels were confirmed using RT-PCR. n = 6. Mean ^+/−^ SEM. *p < 0.05. (**F,G)** Effects of *Flii* gene expression on Axin-2 and β-catenin levels were also confirmed using Western Blotting. Pooled samples, representative blots of repeated experiment. n = 6. Mean ^+/−^ SEM. *p < 0.05. (**H)** Schematic illustration of Flii regulation of activated Wnt/β-catenin signalling pathway. Detailed explanation can be found in the manuscript body.

Flii has previously been shown to modulate Wnt/β-catenin signalling and regulate tissue regeneration^16,17,26^. To determine the effect of differential *Flii* gene expression on regeneration of distal colonic crypts in colitis-induced mice, Wnt/β-catenin signalling was assessed. *Flii* overexpression was found to inhibit Wnt/β-catenin signalling, with significantly decreased levels of Lgr6 receptor and intracellular β-catenin levels while *Flii* deficiency resulted in a significantly decreased number of Axin-2 positive cells (Fig. 6A–E). These findings were further confirmed using PCR and Western Blotting (Fig. 6E–G). These findings suggest that Flii effects on Wnt/β-catenin signalling pathway may underpin the impaired regeneration of colonic crypts observed in *Flii* over-expressing mice (Fig. 6H).

## Discussion

UC is a chronic inflammatory disease that can lead to severe consequences including colectomy and significantly increased risk of colorectal cancer. Retrospective and prospective studies with UC patients have highlighted the importance of mucosal healing as the critical endpoint in disease management^27,28^. This study has shown that human UC lesions have significantly elevated levels of Flii, a cytoskeletal protein previously shown to impair healing responses and to be upregulated in response to tissue inflammation in a number of different inflammatory skin disease conditions including human psoriasis, dermatitis and inflammation mediated epidermolysis bullosa acquisita^20,21,24^. In the current study, Flii was prominent in the inflammatory infiltrate of human lamina propria surrounding the distal colon crypts suggesting its potential involvement in the inflammatory pathway of human colitis. Studies have previously demonstrated Flii expression in organs susceptible to inflammation and fibrosis including liver, lung and kidney^29^. Although Flii levels in normal gut are low, it has previously been shown to be upregulated in response to injury and inflammation hence its involvement in inflammatory mediated conditions like human UC is not surprising^10,20,29^.

This study therefore set out to determine the extent of Flii involvement in UC and mucosal healing using an acute model of DSS-induced colitis which closely resembles clinical and histopathological features of human UC^30^. While the mechanism of DSS-induced damage in the colon remain unclear, damage is most prominent in the distal colon and is believed to be caused by alterations in colonic microflora, direct cytotoxic effects on the epithelium and increased macrophage and neutrophil activity resulting in free radical production^31^. Clear differences in disease severity were observed in response to altered Flii levels including higher degree of colon shortening, decreased crypt depth and increased inflammation in animals with high Flii. In contrast, reducing Flii expression resulted in significantly reduced levels of colon shortening, no evidence of rectal bleeding, significantly decreased disease severity and significantly higher crypt area index compared to mice with normal levels of Flii. Together, these results suggest that high levels of Flii in the gut of patients with UC, may exert a negative influence on clinical disease progression and recurrence.

Acute DSS-induced colitis is characterized by an increase in pro-inflammatory cytokines TNF-α and IFN-γ which are the major proinflammatory cytokines that synergistically drive epithelial barrier dysfunction and apoptosis, particularly during colitis, while chronic DSS-induced colitis comprises focal Wnt/β-catenin mediated epithelial regeneration and both Th_1_ and Th_2_ cytokine profiles^32–34^. While the atypical cytokine profile of a Th_2_ reaction is more prevalent in patients with UC, the absence of prototypic IL-4 combined with Arthurs reaction of neutrophil infiltration contributes to disease chronicity^35^. Additionally, in patients, IFN-γ has been causatively involved in UC epithelial homeostasis and intestinal inflammation^36^ while IL-17A is associated with increased UC disease activity and ability to trigger and amplify multiple inflammatory pathways regulating gut inflammation^37^. Flii has been demonstrated to regulate inflammation through its effects on TLR4 signalling pathway both intracellularly and extracellularly^18,38^. Its intracellular effect on TLR4 signalling and subsequent NF-ĸB secretion is mediated via interactions with Myd88 and has been shown to affect inflammation signalling in inflammatory mediated psoriasiform dermatitis^20^. Flii is secreted through a non-classical late endosome/lysosome mediated pathway by both fibroblasts and macrophages, and is present in both acute and chronic human wound fluids^10^. Like its family member gelsolin, plasma Flii functions to scavenge extracellular actin following injury and mediate inflammatory responses^38,39^. Plasma Flii binding to lipopolysaccharide alters macrophage activation and subsequent macrophage secretion of TNF-α^38^. Additionally, a recent study has shown that Flii alters inflammatory responses in inflammation mediated atopic dermatitis, where high Flii correlates with increased inflammatory responses resulting in a skewed Th_2_ response^24^.

In this study, a significantly increased inflammatory cell infiltrate was observed in the distal colon of colitis-induced *Flii* overexpressing animals compared to controls while colitis-induced mice with low levels of Flii showed significantly decreased MPO activity in the distal colon suggesting Flii may augment UC mediated inflammation and mucosal healing. Furthermore, examining the effect of Flii on cytokines known to drive UC mediated tissue inflammation revealed that reducing Flii expression results in a decrease in tissue inflammation and significantly lower levels of pro-inflammatory cytokines including TNF-α, IL-17A and IL-5; all of which would favour decreased UC disease severity. In contrast, but in agreement with increased UC disease severity observed in *Flii*^*Tg/Tg*^ mice, distal colons of these colitis-induced mice showed an exacerbated immune response with significantly increased expression of Th_1_ and Th_2_ cytokines including TNF-α, IFN-γ, IL-5 and IL-13. Indeed, this atypical Th_2_ response with increased IL-5 and IL-13 levels has been observed in chronic UC patients^40^. The observed effects of Flii on Th_1_/Th_2_ immune responses are also in agreement with previous reports showing high levels of Flii alter immune responses in inflammation mediated conditions including psoriasiform dermatitis and atopic dermatitis^20,24^. Together, these findings suggest that Flii plays an important role in inflammatory mediated conditions, like UC, and that its effect on inflammation promotes a Th_2_ mediated response in UC which would favour more chronic disease state^30^. Additionally, numerous studies to date have postulated that this exacerbated Th_2_ mediated response in UC patients is an attempt to activate mucosal Wnt/β-catenin signalling known to regulate intestinal epithelial stem cell proliferation required for regeneration of colonic crypts^41–43^. Cooperative interaction between Wnt and R-spondin ligands establishes a molecular precedent for regulation of intestinal stem cells required for colonic tissue regeneration^44^. Our recent study has described the Flii regulation of Wnt signalling during skin homeostasis and wound healing indicating that Flii negatively regulates epidermal stem cell activation via its effects of Wnt signalling pathway^45^. How Wnt/β-catenin signalling pathway contributes to wound healing during colitis has yet to be formally established. However, it is well accepted that Wnt signalling pathway is crucial for development and renewal of the intestinal epithelium^46^. Here we demonstrate that *Flii* overexpression leads to inhibition of Wnt signalling with decreased expression of β-catenin and leucine-rich repeat-containing G protein-coupled receptor 6 (Lgr6) receptor required for R-spondin amplification of canonical Wnt signalling. This agrees with previous studies showing that Flii can inhibit Wnt signalling by binding to negative regulators of the Wnt signalling pathway through Dishevelled (Dvl) protein interactions^47^.

We show that Flii overexpression leads to decreased β-catenin expression suggesting that Flii may decrease β-catenin stabilisation and increase ubiquitin-mediated and proteasomal β-catenin degradation. This important finding is in agreement with previous studies which suggested that Flii inhibition of Wnt signalling occurs via β-catenin binding and inhibited lymphoid enhancer factor (LEF) and T-cell factor (TCF) transcriptional factor-mediated expression of Wnt target genes^6^. Tight regulation of Wnt/β-catenin signalling by Flii was also evident in *Flii* deficient mice which showed decreased Axin-2 expression, supporting our earlier findings which suggested that Flii may impact β-catenin via Axin-2 regulation at the transcriptional level^45^. Further studies are required to identify the specific molecular patterns governing Flii involvement in Wnt signalling pathway and subsequent effects on activation and proliferation of intestinal stem cells.

In conclusion, we have demonstrated that Flii is upregulated in the distal colon of human UC patients. High levels of Flii correlate with greater inflammation and exacerbated Th_1_/ Th_2_ immune responses resulting in increased disease severity in mouse models of DSS-induced colitis, while reducing Flii levels promotes decreased gut inflammation and improved mucosal healing. Although the exact mechanisms of Flii function in UC are yet to be elucidated, our results suggest that Flii negatively regulates Wnt/β-catenin signalling required for regeneration of colonic crypts. Together these results suggest that manipulation of Flii levels may lead to potential novel therapeutic interventions by which UC disease severity, tissue inflammation and mucosal healing might be improved.

## Materials and Methods

### Human studies

Colonoscopies were performed at The Queen Elizabeth Hospital (TQEH, Adelaide). Ten adult subjects with UC, and 10 normal adult subjects with non-inflammatory conditions, such as irritable bowel syndrome or who attended for colon cancer screening were included in the study. All experimental protocols were approved by the Human Ethics Committee of the TQEH in accordance with relevant guidelines and regulations. Approval was given to perform additional biopsy for research and to archive biopsies for future studies and written informed consent was obtained from all participants^48^. Colonic biopsies in histologic paraffin blocks were retrieved and histological sections (4 µm) stained with haematoxylin and eosin (H&E) and standard immunohistochemistry staining protocols (see below) for Flii (2 mg/ml; anti-Flightless I sc-30046 rabbit IgG; Santa Cruz Biotechnology, CA, USA) and 4′6-diamidino-2-phenylindole (DAPI; 0.1 mg/ml; D1306; Live Technologies Australia, VIC, AUS) as previously described^20^.

### Animal studies

Female Balb/c mice were maintained according to the Australian Standards for Animal Care under the protocols approved by the Child, Youth and Women’s Health Service Animal Ethics Committee, The University of Adelaide Animal Ethics Committee and University of South Australia Animal Ethics Committee (AEC 962/12/16 and AEC 137a/13). All strains were BALB/c-congenic and were maintained as homozygous colonies or by continuous backcrossing to BALB/c animals. Wild-type controls were obtained from BALB/c inbred litters. The murine alleles of Flii used in this study include: a heterozygous carrier of the murine Flightless I gene (Flii): Flii tm1Hdc (MGI:2179825) written as *Flii*^+/−^; and Tg(FLII)2Hdc (MGI:4939366), a transgenic strain expressing exogenous human flightless I (FLII)^9,49^. *Flii*^+/−^ were generated by loss of function mutation in the *Flii* gene via homologous recombination in embryonic stem cells and passage of these cells through the germ line following chimera production^50^. The generation of *Flii*^+/−^ mice and the resulting mutation are described in detail in Campbell *et al*., (2002) and a diagram of the targeting strategy is illustrated in Supplementary Fig. 2A. The heterozygous mice were identified using three primer PCR sets that amplified products specific to the wild-type or targeted allele as illustrated in Supplementary Fig. 2B. The PCR was performed on DNA extracted from ear biopsies of potential heterozygotes. The animals with one wild-type copy of the *Flii* gene and one mutant copy of the *Flii* gene express no more than 50% of the normal *Flii* gene expression^49^.

Mice homozygous for the transgene were used in this study; had two copies of *Flii* gene and two copies of human *FLII* transgene (*Flii*^+/+^; *Flii*^*Tg/Tg*^) with significantly elevated levels of Flii protein compared to wild-type^50^ and are denoted as *Flii*^*Tg/Tg*^ throughout the article. Mice carrying additional copies of the *Flii* gene were generated by introduction of a cosmid construct into the mouse genome using transgenesis. At the time of strain production, the cosmid contained the human *Flii* gene and the surrounding sequences with the extent of the construct being defined via restriction mapping^50^. The availability of the mouse genome allowed estimation of the extent of the cosmid. Currently, it is known that the cosmid contains all the neighbouring SMCR7 gene and parts of the Topo and LLGL1 genes (Supplementary Fig. 2C). The transgenic strain was backcrossed to BALB/c animals for 10 generations before being intercrossed; and homozygous animals were classified via progeny testing following established protocols^49,50^. The mouse colony was subsequently maintained by intercross of animals homozygous for the transgene. The expression of Human *FLII* gene was examined using species specific RT-PCR showing *FLII* expression in all tissues examined (adult brain, heart, lung, muscle, spleen and skin) (Supplementary Fig. 2D)^50^. An upregulation of Flii protein levels was confirmed using semi-quantitative Western analysis that showed total (mouse + human) protein levels up to 1.52 fold greater than wild-type levles (Supplementary Fig. 2E)^50^.

Colonic inflammation was induced in mice using Dextran Sulphate Sodium (DSS; colitis grade; MW 36,000–50,000; #02160110; MP Biomedicals, Jomar Life Research, SA, AUS)^31^. The DSS induced model of UC has the phenotypic features of human disease including clinical symptoms of diarrhoea, rectal bleeding and weight loss and histological features of ulceration, oedema, crypt and epithelial cell damage, and increased lymphocyte, monocyte and granulocyte infiltration^31^. 2% w/v DSS was introduced into drinking water of *Flii*^+/−^, wild-type and *Flii*^*Tg/Tg*^ mice over a period of seven days^31^. Bodyweights were recorded daily and disease activity index (DAI) was calculated daily from weight loss, general condition, stool consistency and rectal bleeding^31^. On day 7 of the experimental period, mice were euthanized by CO_2_ asphyxiation followed by cervical dislocation and distal colon sections collected for RTq-PCR, immunohistochemistry, MPO analysis and histological processing.

### Histology and immunohistochemistry

Paraffin embedded, fixed tissue samples were stained with H&E or subjected to antigen retrieval and immunohistochemistry following manufacturer’s protocols (DAKO Corporation, DK). H&E stained sections were used for standardised measurements of colon length, crypt depth, crypt area index and histological disease severity following established protocols^25,31^. Briefly, crypt depth and area index were determined in a blinded study using Image-Pro Plus software (Media Cybernetics, MD, USA) and 40 well orientated crypts per tissue per mouse were analysed and a mean value obtained^25^. Histological disease severity was performed semi-quantitatively for 7 parameters including: enterocyte, crypt, and crypt cell disruption, reduction in goblet cells numbers, lymphocytic and polymorphonuclear cell infiltration, and thickening/oedema of the submucosa and muscularis externa^31^. For immunohistochemistry, following blocking in 3% normal goat serum, primary antibodies were applied at 2 mg/ml (4 °C) overnight in a humidified chamber. Primary antibodies included: anti-Flightless I sc-30046 rabbit IgG, anti-PCNA sc-56 mouse IgG2a, and anti-TNF-α sc-52746 mouse IgG, anti-Flightless I sc-21716 mouse monoclonal IgG, anti-β-tubulin sc-51670 mouse monoclonal IgG, and anti-β-catenin sc-7963 rabbit polyclonal IgG all purchased from Santa Cruz Biotechnology, CA, USA. Isotype control mouse IgG2a antibody (ab170191) and anti-Axin-2 (ab32197) rabbit polyclonal IgG were purchased from Abcam, VIC, AUS. Species specific secondary antibodies used included Alexa Fluor goat anti-rabbit 488 (A11006), goat anti-mouse 633 (A21050), goat anti-mouse 488 (A11001) and goat anti-rabbit 633 (A31577) which were purchased from Life Technologies Australia, VIC, AUS. The nuclei were counterstained with 4′6-diamidino-2-phenylindole (DAPI; 0.1 mg/ml; D1306; Live Technologies Australia, VIC, AUS) for 3 min at room temperature prior to mounting sections in Fluorescence Mounting Medium (DAKO Corporation, DK). Images were captured on an Olympus microscope and CellSense Live Science Imaging Software program (Olympus, Germany) was used to determine the integrated fluorescence intensity. Negative controls and isotype control antibody were included to demonstrate antibody staining specificity. Control samples underwent the same staining procedure outlined except the primary or secondary antibody was omitted. All control sections had negligible immunofluorescence.

### Myeloperoxidase assay

Myeloperoxidase assay (MPO) was performed to detect neutrophil infiltration using protocols previously published^51^. Briefly, distal colon tissue was homogenized and centrifuged at 13,000 g for 12 min, supernatant was discarded and 0.5% hexadecyltrimethyl ammonium bromide buffer (Sigma Aldrich, NSW, AUS) was used to re-suspend the tissue homogenate. After vortexing and centrifuging for 2 min, water control and test samples were aliquoted (50 µls) into duplicate wells of the 96-well plate. A reaction solution of 4.2 mg of O-dianisidine dihydrochloride reagent, 12.5 µls H_2_O_2,_ 2.5 mL potassium phosphate buffer (pH 6.0), 22.5 mL distilled water was added to each well (200 µls) and absorbance was measured at 450 nm at 1 min intervals for 15 min using a spectrophotometer (Victor X4 Multilabel Reader, Perkin Elmer, SGP).

### Q-PCR

Harvested tissue was snap-frozen in liquid nitrogen and total RNA was isolated from 1 cm of distal colon per sample (n = 6/genotype) using Ultraclean Tissue and Cell RNA Isolation Kit (MoBio Laboratories, CA, USA) according to the manufacture’s protocol. Total cDNA was reverse-transcribed from equal amount of RNA (200 ng) per sample using iScript cDNA synthesis kit (Bio-Rad Laboratories, CA, USA) according to manufacturer’s protocol. The PCR reaction mix consisted of 2 μl RT reaction mix, 5 μl 5×PCR buffer, RNA and water making a total volume of 20 μl. The reaction was initiated by incubation at 25 °C for 5 min, followed by annealing at 42 °C for 30 min and final incubation at 85 °C for 5 min followed by 10 min at 4 °C. Quantitative PCR was performed using iQ SYBR Green Supermix (Bio-Rad Laboratories, CA, USA) in triplicate reactions in CFX connect real-time PCR system and analysed by CFX Maestro software (Bio-Rad Laboratories, CA, USA). Each Q-PCR reaction mix consisted of 10 μl supermix, 1 μl of cDNA, primers and water making a total volume of 20 μl. A three-step PCR was carried out with initial denaturation for 30 s at 95 °C, followed by 39 cycles of denaturation for 5 s at 95 °C and annealing for 20 s at 60 °C with a final extension of denaturation for 10 s at 95 °C and annealing for 5 s at 605 °C. CyPA and GAPDH were used as reference genes and the inter-reaction calculator method was applied for all plates. For relative comparison, the cycle threshold value (Ct) was analysed using the ΔΔCt method and data reported as Ct normalized to reference genes. Gene expression was expressed as fold change of WT value. Sequences for PCR primers are listed in Supplementary Table 1.

### Western blotting

Protein was extracted from distal colon tissue sections of colitis-induced *Flii*^+/−^, WT and *Flii*^*Tg/Tg*^ mice by homogenising tissue in a lysis buffer (50 mM Tris pH 7.5, 1 mM EDTA, 50 mM NaCl, 0.5% Triton X-100) containing protease inhibitor tablet (1 per 10 ml; Complete, Mini (Roche, Australia). Samples were centrifuged, and supernatants collected. BCA kit was used to quantify protein levels and 50 µg of protein was run on 10% SDS-PAGE gels at 100 V for 1 hour and transferred to nitrocellulose membrane using standard Towbins Buffer with 20% Methanol at 100 V for 1 hour. Following blocking in 12% milk-blocking buffer for 15 minutes. Primary antibodies including anti-β-catenin sc-7963 rabbit polyclonal IgG (1:400), anti-Axin-2 (ab32197) rabbit polyclonal IgG (1:400) and anti-β-tubulin sc-51670 mouse monoclonal IgG (1:3000) were diluted in buffer and applied to the membrane at 4 °C overnight. Species-specific secondary horseradish peroxidase-conjugated antibodies were diluted in 5% milk-blocking buffer and applied to the membrane at room temperature for 1 hour. Protein bands were detected using Super Signal West Femto (Pierce Biotechnology, Rockford, IL) and visualized with GeneSys analysis software (Syngene, MD).

### Statistical analysis

Parametric data were expressed as mean ± standard error of the mean (SEM). Histological crypt depth and MPO activity were analysed using a one-way ANOVA with Tukey’s post hoc tests. Disease activity index were analysed using repeated measures ANOVA with least significance difference to compare the differences both between and within groups. Non-parametric data included histological damage severity scores and were analysed using a Kruskal Wallis test with Mann Whitney U tests, expressed as median range. p < 0.05 was considered statistically significant.

## Data Availability

All data sets generated during and/or analysed during the current study are available from the corresponding author on reasonable request.

## Supplementary information

Supplementary Matrerial
