# Supplementary material for: Flightless I exacerbation of inflammatory responses contributes to increased colonic damage in a mouse model of dextran sulphate sodium-induced ulcerative colitis
Source: Sci Rep. 2019 Sep 5;9:12792. doi: 10.1038/s41598-019-49129-6 (PMC6728368; doi:10.1038/s41598-019-49129-6)
Supplement: Supplementary file 1 — Supplementary Matrerial [file 41598_2019_49129_MOESM1_ESM.pdf]

Flightless I exacerbation of inflammatory responses contributes to increased colonic damage in a mouse model of dextran sulphate sodium-induced ulcerative colitis

Kopecki Z.[1,2* #], Yang G.[1], Treloar S.[3], Mashtoub S.[4,5], Howarth G.S.[2], Cummins A.G.[6] and Cowin A.J.[1,2 #]

[1]Regenerative Medicine, Future Industries Institute, University of South Australia, Mawson Lakes, Adelaide, South Australia, Australia; [2]School of Pharmacy and Medical Sciences, University of South Australia, Adelaide, South Australia, Australia; [3]School of Animal and Veterinary Sciences, The University of Adelaide, Roseworthy, Adelaide, South Australia, Australia; [4]Department of Gastroenterology, Women's and Children's Hospital, North Adelaide, South Australia, Australia; [5]Discipline of Physiology, Adelaide Medical School, The University of Adelaide, Adelaide, South Australia, Australia; [6]Department of Gastroenterology and Hepatology, The Queen Elizabeth Hospital, Woodville South, Adelaide, South Australia; Australia.

***Correspondence:** Dr Kopecki Z. Regenerative Medicine, Future Industries Institute, University of South Australia. Tel +61 8 83026384, Fax +61 8 83025639. Email: zlatko.kopecki@unisa.edu.au

[#] Dr Kopecki and Prof Cowin contributed equally to the direction of the study.

Supplementary Material:

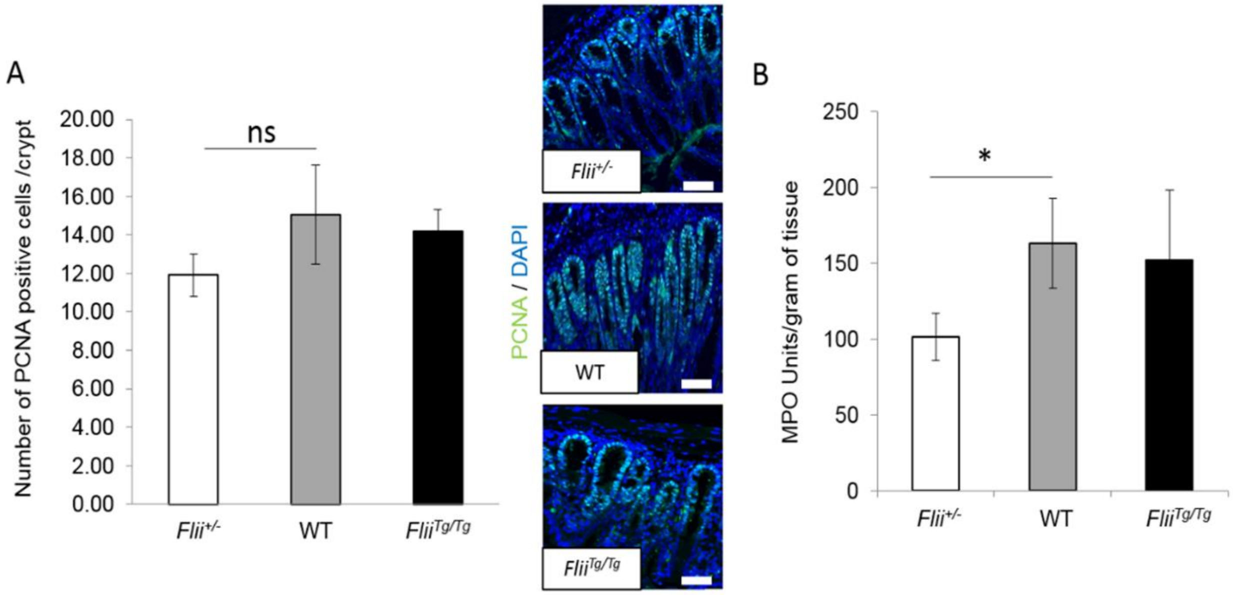

Supplementary Figure 1. Decreasing *Flii* reduces pro-inflammatory cell infiltrate in a DSS model of UC. A Altering *Flii* expression has no effect on enterocyte proliferation in the DSS model of UC. **B** Decreasing *Flii* levels significantly reduces the myeloperoxidase levels in mucosal tissue indicative of significantly reduced pro-inflammatory cell infiltrate in colitis-induced *Flii* deficient mice compared to wild-type controls. n=8/genotype. Magnification x10. Scale Bar = 100µm. Mean +/- SEM. *$p<0.05$.

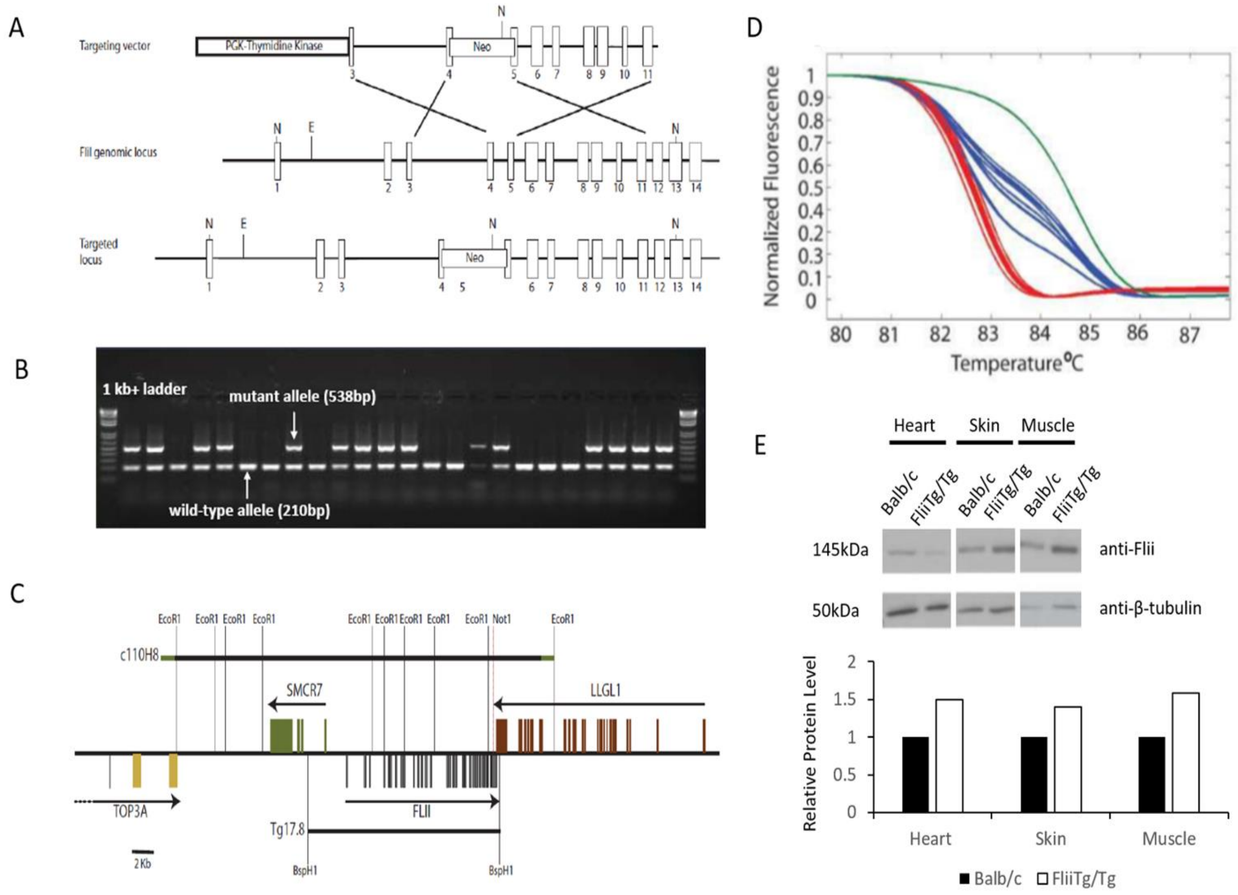

Supplementary Figure 2. Targeted disruption of the *Flii* gene and generation of *Flii* transgenic mice with ubiquitous overexpression of *Flii* gene. A Schematic representation of the domain structure of the targeting vector, relevant portion of the *Flii* gene and the targeted allele after homologous recombination. Restriction enzymes sites, *BspEI* is denoted by B, *EcoRV* by E and *NcoI* by N. *Flii* exons are represented by the numbered open boxes. The tk-neo and pgk-thymidine kinase casettes are indicated. **B** Three primer PCR indicating wild-type (210bp) and mutant allele (538bp) products. Animals with one wild-type copy of the Flii gene and one mutant allele expressed no more than 50% of the normal wild-type Flii expression levels. Figure adapted from [48]. **C** Domain structure of the cosmid containing Flii gene which is used to generate Flii transgenic mice. Cosmid contains Flii gene, SMCR7 gene, parts of TOP3A and LLGL1 genes. Restriction sites on the cosmid are also illustrated. Figure adapted from [48]. **D** Characterization of the *Flii*$^{Tg/Tg}$ mouse strain using HRM analysis of species-specific products. Green – Melt curve of human *FLII* amplicon obtained by PCR of genomic DNA; Red – Melt curve of the mouse Flii amplicon following PCR of cDNA from six tissues (brain, heart, lung, muscle, skin and spleen) of BALB/c animal; Blue – analysis of amplicons obtained by PCR of cDNA from six tissues of *Flii*$^{Tg/Tg}$ animal. The melt curve is intermediate between the mouse (red) and human (green) amplicons indicating that the cDNA contains a mixture of two species. Figure adopted from [49]. **E** Western analysis of total Flii protein in heart, skin and muscle of BALB/c wild-type mice and age and sex matched *Flii*$^{Tg/Tg}$ counterparts illustrating clear Flii overexpression in transgenic mice. Figure adopted from [49].

Primers	Sequence 5'-3'
CyPA F	GGTTGGATGGCAAGCATGTG
CyPA R	TGCTGGTCTTGCCATTCCTG
GAPDH F	GGGCTCTCTGCTCCTCCCTGT
GAPDH R	CGGCCAAATCCGTTCACACCG
TNFα F	GCCCACGTCGTAGCAAACCAC
TNFα R	GCAGGGGCTCTTGACGGCAG
IL-17A F	CTGCTGAGCCTGGCGGCTAC
IL-17A R	CATTGCGGTGGAGAGTCCAGGG
IFNγ F	AAAGAGATAATCTGGCTCTGC
IFNγ R	GCTCTGAGACAATGAACGCT
IL-5 F	AGCACAGTGGTGAAAGAGACCTT
IL-5 R	TCCAATGCATAGCTGGTGATTT
IL-13 F	TGGGTCCTGTAGATGGCATTG
IL-13 R	AGACCAGACTCCCCTGAGCA
Axin-2 F	TAGTCCCAGAGCCCGTCACAG
Axin-2 R	GAACGCTGGCAGACAGGACATA
Lgr6 F	CTGATGCACCTGAAGCTCAA
Lgr6 R	ACAGCACTGGTAGGCGTAGG

Supplementary Table 1. Q-PCR primers used in this study.
